# Effect of the COVID-19 Lockdown on Mobile Payments for Maternal Health: Regression Discontinuity Analysis

**DOI:** 10.2196/49205

**Published:** 2024-07-30

**Authors:** Samuel Knauss, Gracia Andriamiadana, Roxane Leitheiser, Zavaniarivo Rampanjato, Till Bärnighausen, Julius Valentin Emmrich

**Affiliations:** 1 Global Digital Last Mile Health Research Lab Charité Center for Global Health Charité - Universitätsmedizin Berlin, Germany Berlin Germany; 2 Heidelberg Institute of Global Health Medical Faculty and University Hospital University of Heidelberg Heidelberg Germany; 3 Wolfson Institute of Population Health Queen Mary University of London London United Kingdom; 4 Ministry of Public Health of the Republic of Madagascar Antananarivo Madagascar; 5 Harvard Center for Population and Development Studies Harvard University Cambridge, MA United States; 6 Africa Health Research Institute Mtubatuba South Africa

**Keywords:** digital health, behavioral surveillance, digital health wallet, mobile money, COVID-19, health financing, public health, sub-Saharan Africa

## Abstract

**Background:**

The COVID-19 pandemic resulted in the unprecedented popularity of digital financial services for contactless payments and government cash transfer programs to mitigate the economic effects of the pandemic. The effect of the pandemic on the use of digital financial services for health in low- and middle-income countries, however, is poorly understood.

**Objective:**

This study aimed to assess the effect of the first COVID-19 lockdown on the use of a mobile maternal health wallet, with a particular focus on delineating the age-dependent differential effects, and draw conclusions on the effect of lockdown measures on the use of digital health services.

**Methods:**

We analyzed 819,840 person-days of health wallet use data from 3416 women who used health care at 25 public sector primary care facilities and 4 hospitals in Antananarivo, Madagascar, between January 1 and August 27, 2020. We collected data on savings, payments, and voucher use at the point of care. To estimate the effects of the first COVID-19 lockdown in Madagascar, we used regression discontinuity analysis around the starting day of the first COVID-19 lockdown on March 23, 2020. We determined the bandwidth using a data-driven method for unbiased bandwidth selection and used modified Poisson regression for binary variables to estimate risk ratios as lockdown effect sizes.

**Results:**

We recorded 3719 saving events, 1572 payment events, and 3144 use events of electronic vouchers. The first COVID-19 lockdown in Madagascar reduced mobile money savings by 58.5% (*P<.*001), payments by 45.8% (*P<*.001), and voucher use by 49.6% (*P<*.001). Voucher use recovered to the extrapolated prelockdown counterfactual after 214 days, while savings and payments did not cross the extrapolated prelockdown counterfactual. The recovery duration after the lockdown differed by age group. Women aged >30 years recovered substantially faster, returning to prelockdown rates after 34, 226, and 77 days for savings, payments, and voucher use, respectively. Younger women aged <25 years did not return to baseline values. The results remained robust in sensitivity analyses using ±20 days of the optimal bandwidth.

**Conclusions:**

The COVID-19 lockdown greatly reduced the use of mobile money in the health sector, affecting savings, payments, and voucher use. Savings were the most significantly reduced, implying that the lockdown affected women’s expectations of future health care use. Declines in payments and voucher use indicated decreased actual health care use caused by the lockdown. These effects are crucial since many maternal and child health care services cannot be delayed, as the potential benefits will be lost or diminished. To mitigate the adverse impacts of lockdowns on maternal health service use, digital health services could be leveraged to provide access to telemedicine and enhance user communication with clear information on available health care access options and adherence to safety protocols.

## Introduction

Globally, more than 6.9 million people have died from COVID-19, and the number of COVID-19–related deaths is still steadily increasing (data as of December 2023) [1]. Beyond the direct effect of the disease, the pandemic continues to disrupt essential health services worldwide, undermining the achievements of existing public health programs [2]. Health systems and health service delivery were extensively disrupted by COVID-19 in sub-Saharan Africa (SSA) [3]. Maternal health services were among the most severely affected health services in SSA [4]. Redeployment of health staff to provide COVID-19 relief, supply chain interruptions, drug price surges, and pandemic control measures like lockdowns further hampered access to maternal care [5].

Within less than a generation, mobile communication and mobile payments, also known as mobile money, have become ubiquitous in SSA [6]. The COVID-19 pandemic resulted in an unprecedented increase in the popularity of this technology as a safer and more efficient alternative to cash payments, particularly for unbanked populations. Several countries in SSA promoted the use of mobile money during the pandemic through a variety of regulatory and policy-led measures, including fee waivers, increased transaction limits, humanitarian cash transfers, and flexible registration of new users [7]. However, the pandemic also had negative effects on the use of mobile money. More than half of mobile money users in Kenya and Mozambique reported difficulties in depositing or withdrawing funds from a cash-in/cash-out agent [7].

Mobile money is being used routinely in the health sector in many countries in SSA, improving financial risk protection and access to essential health services [8]. Examples in SSA include remote health insurance enrollment, premium payment, and reimbursement [9]; humanitarian cash transfers and electronic health vouchers [10]; and credits and loans [8]. Overall, mobile money users have a lower risk of catastrophic health expenditure (defined as out-of-pocket expenses exceeding 10% of total income or consumption, or exceeding 40% of nonfood spending) during emergency care and are less likely to reduce nonmedical expenses for education or food than nonusers [11]. However, the effects of lockdown measures on mobile payments for health in SSA are not clear.

In Madagascar, a nation of 25 million people, financial barriers significantly impede access to maternal health care [12]. Fewer than half of expectant mothers adhere to the World Health Organization’s recommendation of completing 4 antenatal care visits, and over half of births occur without skilled personnel. In 2017, the maternal mortality ratio stood at 335 per 100,000 live births, with figures potentially tripling in the poorest regions [13]. Out-of-pocket payments account for nearly a quarter of health care spending. Despite efforts toward universal health coverage incorporating mobile technologies, specialized mobile money services for maternal health savings or insurance have not been established on a national level. The probability of incurring impoverishing expenses during pregnancy remains substantial [14,15]. Nevertheless, mobile phone subscriptions have soared from under 3 per 100 people in 2005 to 40 per 100 people in 2018, with mobile money accounts surpassing traditional banking figures in 2015 [16]. Human-centered mixed-methods research in the capital Antananarivo aimed to identify the framework and user experience for a mobile-based service for maternal health care payments and savings [17-19], and subsequently, a mobile maternal health wallet (MMHW) was developed and implemented in the capital, revealing a strong perceived value of mobile money for maternal health, particularly among women from lower-income backgrounds [17].

Our study aimed to demonstrate the causal effect of COVID-19 lockdowns on the use of digital health services and explore age-dependent differential effects. In the context of maternal health in Madagascar, we examined 3 primary uses of the MMHW: savings, payments, and voucher use for maternal care. Reductions in savings suggest that lockdowns lowered women’s future health care expectations, while declines in payment and voucher use indicate decreased actual health care utilization. This is significant since many maternal and child health care services cannot be delayed without losing or diminishing potential benefits. Our study did not intend to assess the overall impact of the COVID-19 pandemic on health care utilization or mobile money use. Rather, our focus was on examining how lockdowns affect the utilization of digital health services for maternal care, specifically in terms of mobile payments for maternal health care services. These findings can guide health policy in developing strategies to mitigate lockdown-related challenges in the future.

## Methods

### Study Setting

We conducted this study in 25 public sector primary care facilities and 4 referral hospitals in 3 administrative districts of Antananarivo, the capital of Madagascar.

Participating primary care facilities are typically staffed by at least one doctor and a couple of midwives or nurses supported by 15-30 community health workers who promote antenatal care and provide education on health, pregnancy complications, and nutrition. The districts are urban, periurban, and rural. All health facilities participating in the study remained open during the lockdown. The study region had 2.2 million inhabitants, 31% of whom lived under the national poverty line of US $1.90 in 2019 (2011 purchasing power parity), considerably less than the Malagasy average (71%) [20]. In the study region, around 65% of pregnant women complete at least 4 antenatal care visits, 68% deliver in a health facility, and 74% receive skilled birth assistance [21]. While antenatal care services and delivery assistance, including cesarean sections, are provided free, patients must cover the costs of medications, tests, and materials. A normal delivery at a participating facility costs around US $12 and a cesarean section costs around US $128, equating to 3% and 32% of the average annual salary in the study region, respectively [22].

### Study Design

**Figure 1:**
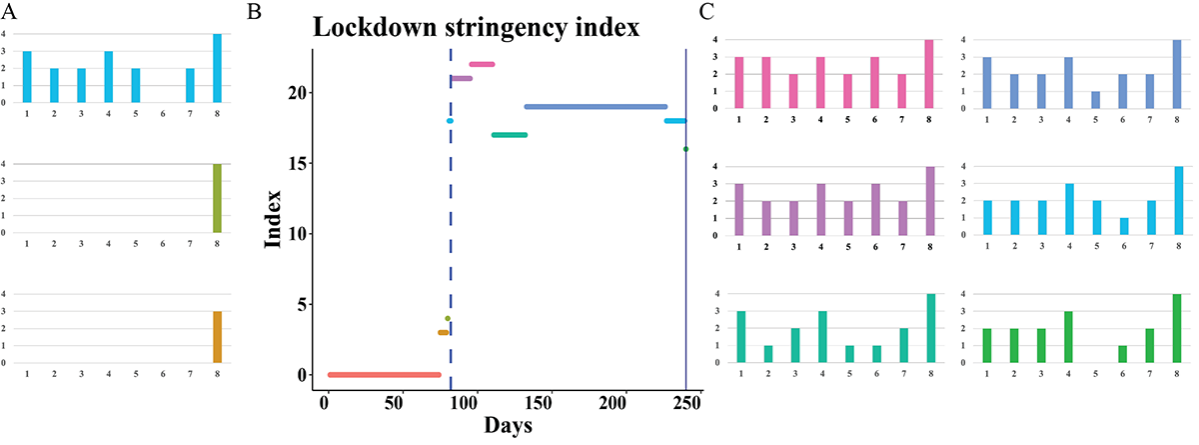
Overview of the lockdown stringency index for Madagascar according to containment and closure policy indicators from the Oxford COVID-19 Government Response Tracker. Panel B displays the overall lockdown stringency index, a composite measure based on several policy indicators, during the study period. The blue dashed vertical line represents the initiation of lockdown, while the solid blue line signifies its conclusion. Color-coded horizontal lines in panel B correspond to the specific periods for which 8 containment and closure policies are detailed in the respective bar charts in panels A (before lockdown) and C (during lockdown). Containment and closure policies are listed on the x-axis of each subpanel and rated on a scale from 0 (least severe restriction) to 4 (most severe restriction) according to the Oxford COVID-19 Government Response Tracker on the y-axis. The 8 policies are as follows: (1) school closure, (2) workplace closure, (3) canceling of public events, (4) restrictions on gatherings, (5) public transport closure, (6) stay-at-home requirements, (7) restrictions on internal movement, and (8) international travel controls.

To estimate the effect of the lockdown on savings, payments, and electronic voucher use for maternal health care, we used a quasiexperimental regression discontinuity design. Regression discontinuity designs are considered the most robust methods to estimate causal effects when random assignment is not feasible [23]. They can be implemented when treatment is assigned based on a cutoff value of a continuous running variable. We used the count of consecutive days starting from the beginning of the study period on January 1, 2020, as our running variable. The cutoff value for lockdown onset was based on data from the Oxford COVID-19 Government Response Tracker (OxCGRT) [24]. We calculated a containment stringency index ranging from 0 to 23 using all “containment and closure policy” indicators included in the OxCGRT data set. We obtained data for Madagascar from the OxCGRT for the study period from January 1 to August 27, 2020, as the period covering the first lockdown in Madagascar. The lockdown came into effect on March 23, 2020. During the prelockdown period between January 1, 2020, and March 22, 2020, the median stringency index was 0. During the lockdown period between March 23, 2020, and August 27, 2020, the median stringency index was 19 (Figure 1) [24]. We defined the first day of the lockdown on March 23, 2020 (day 82) as the cutoff value in our regression discontinuity analysis.

### MMHW Intervention

The mobile health wallet was implemented in the study region as part of the intervention package of the 4MOTHERS trial, which has been described previously [25]. The MMHW intervention was implemented by a nongovernmental organization (NGO) in partnership with the Malagasy Ministry of Health’s universal health coverage program. Implementation of the intervention package commenced sequentially at the participating health care providers starting in January 2019. In brief, the MMHW allows users, namely, pregnant women, to save and pay for health care services using mobile money and to receive electronic vouchers that can be redeemed at participating health care providers at the point of care. Electronic vouchers were for antenatal care drugs, emergency referrals, and obstetric ultrasounds at no cost. The MMHW operates on a USSD (Unstructured Supplementary Service Data) menu accessible via GSM (Global System for Mobile Communications) networks provided by the 2 primary mobile phone operators in Madagascar. Users can receive messages and access the service by dialing a 3-digit code followed by the hash sign, without the need for an active internet connection or a smartphone. Users can save to the mobile health wallet using their own mobile money credit or by receiving remittances via mobile money (eg, from relatives and friends). Savings typically require users to convert cash into mobile money with the help of a cash-in/cash-out agent. Mobile money is then saved to the mobile health wallet electronically by the user. At the point of service, health care facility staff use a web-based interface to initiate and authenticate payments or the use of electronic vouchers. This process involves entering necessary treatment details and submitting corroborative documents like invoices or photographs. The staff members of the implementing NGO check and validate each transaction to ensure remittances from the MMHW are only used for maternal care. Potential users learn about the MMHW intervention through media campaigns, community health workers, and health care providers. Registration to the MMHW is performed by employees of the implementing NGO, community health workers, and health care providers, who have received specific training on the sensitization and registration of the MMHW. All pregnant women in the catchment area of a participating health care facility are encouraged to register for the service. For registration, users need to possess an individual SIM card. Users of the MMHW must be able and willing to give verbal consent to the terms and conditions of the MMHW intervention, including the use of anonymized user data for research.

### Participants and Sample Size

We included anonymized data from all women who used the MMHW at least once to save, pay, or redeem an electronic voucher for maternal health care at a participating health care provider during the study period. Health facilities contributing data to the analysis were randomized as part of the main trial, reducing the risk of selection bias and ensuring high transferability of the results. For each of our 3 outcomes (savings, payments, and electronic voucher use), we observed 3416 women over 240 days, resulting in the observation of 819,840 person-days for each of the outcomes. Because enrollment of participants was performed before the lockdown, it can be assumed that participants’ characteristics did not systematically change at the lockdown. To analyze the differential impact of the lockdown on savings, payments, and electronic voucher use, we divided our population into 3 age groups. The categorization of age groups was based on evidence of the influence of maternal age on health-seeking behavior in the literature [26]. The category “under 25” was chosen to represent younger individuals likely at the beginning of their reproductive years, who may have distinct challenges such as limited financial resources or decision-making autonomy. The “25 to 30” category captured individuals who may be more established in their reproductive life and may potentially have different accesses to resources. The “above 30” category likely included women with greater experience and potentially different familial or economic circumstances.

### Outcomes

The primary outcomes of the study were as follows: (1) saving transactions recorded per user per day (binary variable), (2) payments per user per day (binary variable), and (3) electronic voucher use per user per day (binary variable). Savings were defined as credit transactions to the MMHW. Saving transactions per user were unrestricted. To reflect active use rather than the level of use, we binarized counts per user per day by creating a dummy variable and assigning a value of “1” if any saving transaction was recorded on a given day and a value of “0” if no transaction was recorded. Payments were defined as debit transactions to the MMHW for delivery, including surgical delivery and cesarean section, validated by a staff member of the implementing NGO. Since we only included payments for delivery in our analysis, each user had a maximum of 1 payment during the study period. Electronic voucher use was defined as the recording of a voucher redemption, which was validated by a staff member of the implementing NGO. The mobile health wallet system limited the electronic voucher use to a maximum of 1 voucher per user per day.

### Data Sources and Data Management

Data on savings, payments, electronic voucher use, and demographics were collected from the MMHW online application using a web-based interface available to community health workers and health care facility staff. Demographics were collected during registration, while savings, payments, and voucher use were automatically recorded in the MMHW system in real time upon user or health care worker triggers, yielding high temporary resolution and completeness of the data. All data were cleaned and validated by the staff of the implementing NGO. Data were securely transmitted and stored on the MMHW database adhering to industry standards for data availability, encryption, and protection of health data. For the purpose of this study, data were extracted from the database and fully anonymized by the NGO before being transmitted securely to the research team. We extracted anonymized user identifiers and timestamps for each outcome to convert them into daily binary outcomes per user. As we only included data from users with at least one event for savings, payments, or voucher use during the study period and all days for which no event was recorded were coded as 0, there were no missing data. All data used for this research were kept in a secure password-protected online repository and were accessible only to the investigators.

### Statistical Analysis

We used descriptive statistics to summarize demographics and create crude summaries of outcomes before and during the lockdown. Our primary analysis tested the null hypothesis that the lockdown had no effect on mobile health wallet use. We employed a regression discontinuity analysis as a quasiexperimental approach, treating the start of the COVID-19 lockdown as a natural experiment. Regression discontinuity designs are among the strongest possible designs when random participant assignment is not feasible and are increasingly used in public health research. By comparing MMHW use just before and immediately after the lockdown date, the regression discontinuity analysis allowed us to isolate and measure the causal effect of the lockdown on the use of digital financial services for maternal health under the assumption that all circumstances are similar in all aspects, except for the lockdown itself. We evaluated the relationship between our outcomes and our running variable (consecutive days), allowing for discontinuity at lockdown onset and different slopes on either side of the threshold. Incidence rate ratios (IRRs) and predicted incidence rates along the running variable were estimated by fitting a Poisson regression model with robust standard errors using sandwich estimation for binary outcomes [27]. The Poisson regression model was deemed appropriate after confirming the absence of overdispersion in the data. The model’s goodness-of-fit was further supported by residual deviance values that were consistent with the degrees of freedom [27]. This appropriateness was substantiated by a low frequency of events (ie, payments) relative to the size of the study population and the number of observation periods, which aligns with the assumptions of the Poisson model. The model included a continuous time variable, a binary lockdown variable, and a time-lockdown interaction term. Baseline IRRs at the commencement of the study period were obtained from the intercept of the model, with the running variable set to 0.

A key choice in the design of regression discontinuity analyses is the bandwidth governing the window of data included in the analysis. While a wider window improves the precision of the estimates, it also increases the risk of bias and the risk of introducing unknown confounders in the model. We relied on the data-driven Imbens-Kalyanaraman optimal bandwidth selector to avoid conscious and unconscious biases in the bandwidth selection [28]. The running variable (consecutive days) was centered at the lockdown. We estimated daily trends during the lockdown for each outcome by adding the coefficients associated with time and the time-lockdown interaction. To determine whether there were age-specific differences in the recovery of primary outcomes after the onset of the lockdown, we analyzed each age group separately using the same methods as described above and determined whether the predicted incidence rates recovered by calculating the point at which the extrapolated predicted incidence rate after the lockdown crossed the extrapolated prelockdown counterfactual for each outcome measure.

Statistical significance was set at *P*<.05. No post-hoc analysis was conducted based on our primary results. We conducted a sensitivity analysis for varying bandwidths (±20 days) to check robustness. The results represent intention-to-treat effects on an individual user level. All analyses were performed on a participant level using R 4.0.5 (R Foundation for Statistical Computing) and RStudio Version 1.4.1106 (RStudio Team).

### Ethical Considerations

This was an ancillary study using data from the 4MOTHERS trial, a randomized hybrid effectiveness implementation trial quantifying the effect of a mobile health wallet intervention on maternal health outcomes in Antananarivo (German Clinical Trials Register; DRKS-ID: DRKS00014928). The study was approved by the institutional review board of the University of Heidelberg on February 3, 2020 (reference number: S-428/2019). The funders had no role in the study design, data collection, analysis, interpretation, or writing of the paper. 

## Results

### Participant Characteristics

**Figure 2:**
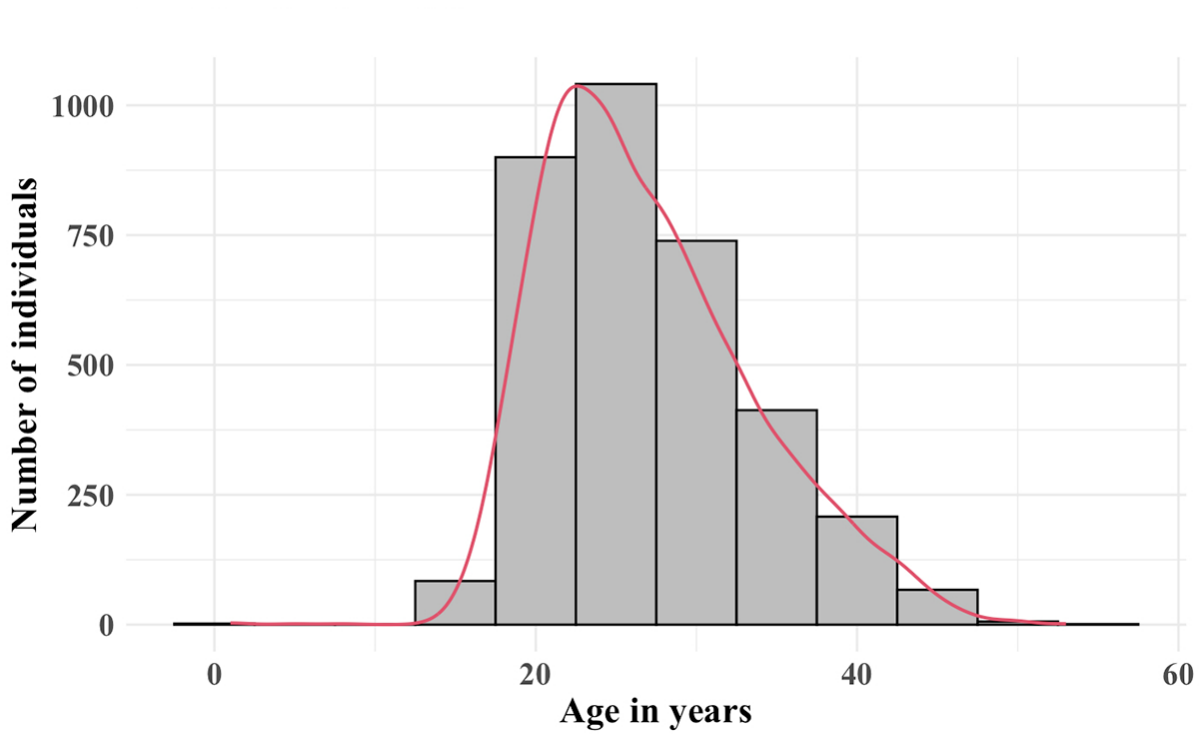
Age distribution of 3416 women using the mobile maternal health wallet to save and pay for health care in Antananarivo, Madagascar, from January 1 to August 27, 2020, who were included in a regression discontinuity analysis to determine the effect of the COVID-19 lockdown on mobile payments for maternal health. Each histogram bar represents the count for a 5-year age range, and the red curve represents the density scaled to fit the count axis.

We analyzed the effect of a national lockdown on the use of a mobile health wallet for maternal health care between January 1, 2020, and August 27, 2020, in Antananarivo, Madagascar, using modified Poisson segmented regression models. Data from 3416 women were included in the study. During the study period, we recorded 3719 saving events, 1572 payment events, and 3144 uses of electronic vouchers. The median age of the study population was 26 years (IQR 9). Figure 2 shows the age distribution. Descriptive statistics of the outcomes for the prelockdown and lockdown are summarized in Multimedia Appendix 1.

### Savings for Pregnancy-Related Health Care Were Reduced During the Lockdown

For the optimal bandwidth of 110 days, we found a significant 58.5% decrease in savings at the lockdown (IRR 0.4152, 95% CI 0.3589-0.4803; *P<.*001) (Table 1). This means that the likelihood of savings for pregnancy-related health care was only 41.5% of the rate before the lockdown, signifying a substantial reduction in women’s ability or willingness to set aside funds for expected maternal health services. This finding remained stable in our sensitivity analysis when varying the bandwidth between 90 and 130 days (Multimedia Appendix 2). During the prelockdown, daily savings increased by 1.2% (IRR 1.0124, 95% CI 1.0098-1.0149) per day. This trend remained stable during the lockdown at 1.1% per day (IRR 1.0113, 95% CI 1.0099-1.0128; *P*=.49) and did not cross the extrapolated prelockdown counterfactual. This indicates that after a pronounced drop at the lockdown, the number of participants saving for health care did not recover rapidly.

**Table 1:** Incidence rate ratios of savings, payments, and electronic voucher use for discontinuity at the lockdown and daily trends during the prelockdown and lockdown among study participants (3416 women; 819,840 events for each use type).

Variable	Incidence rate ratio at baseline (January 1, 2020), value (95% CI)	Incidence rate ratio at the lockdown, value (95% CI)	Incidence rate ratio per day (prelockdown), value (95% CI)	Incidence rate ratio per day (lockdown), value (95% CI)
Savings	0.0090 (0.0082-0.0101)	0.4152 (0.3589-0.4802)	1.0124 (1.0098-1.0149)	1.0113 (1.0099-1.0128)
Payments	0.0026 (0.0021-0.0032)	0.5412 (0.3929-0.7456)	1.0111 (1.0040-1.0183)	1.0054 (0.9979-1.0130)
Electronic voucher use	0.0051 (0.0045-0.0057)	0.5047 (0.4178-0.6095)	1.0034 (1.0005-1.0064)	1.0067 (1.0032-1.0102)

### Payments for Pregnancy-Related Health Care Were Reduced During the Lockdown

Using the optimal bandwidth of 55 days, we identified a drop in payments by 45.8% at lockdown (IRR 0.5412, 95% CI 0.3929-0.7456; *P<.*001). This means that the likelihood of a woman paying for pregnancy-related treatment was at only 54.1% of the prelockdown value, indicating a significant reduction in the use of the MMHW to pay for pregnancy-related health care at participating health facilities. Sensitivity analysis confirmed a robust effect ranging from a 34.8% to 56.3% reduction for bandwidths between 35 and 75 days (Multimedia Appendix 2). During the prelockdown, daily payments increased by 0.54% (IRR 1.0054, 95% CI 0.9979-1.0130) per day. This trend reduced to 0.54% (IRR 1.0054, 95% CI 0.9979-1.0130, *P=*.28) during the lockdown and did not cross the extrapolated prelockdown counterfactual.

### Electronic Voucher Use for Pregnancy-Related Health Care Decreased

At the lockdown, electronic voucher use dropped by 49.6% (IRR 0.5047, 95% CI 0.4178- 0.6095; *P*<*.*001) using the optimal bandwidth of 70 days. This means that the likelihood of a woman using a voucher for pregnancy-related health care was at only 50.4% of the value before the lockdown, implying a substantial decrease in the use of antenatal care at participating facilities. Sensitivity analysis for bandwidths ranging from 50 to 90 days revealed robust results (Multimedia Appendix 2). Voucher use increased by 0.3% per day (IRR 1.0034, 95% CI 1.0005-1.0064) during the prelockdown and increased to 0.67% per day during the lockdown (IRR 1.0067, 95% CI 1.0032-1.0102; *P=*.16), recovering to the extrapolated prelockdown counterfactual after 214 days.

### Recovery of the Use of the Mobile Health Wallet was Dependent on Age

**Figure 3:**
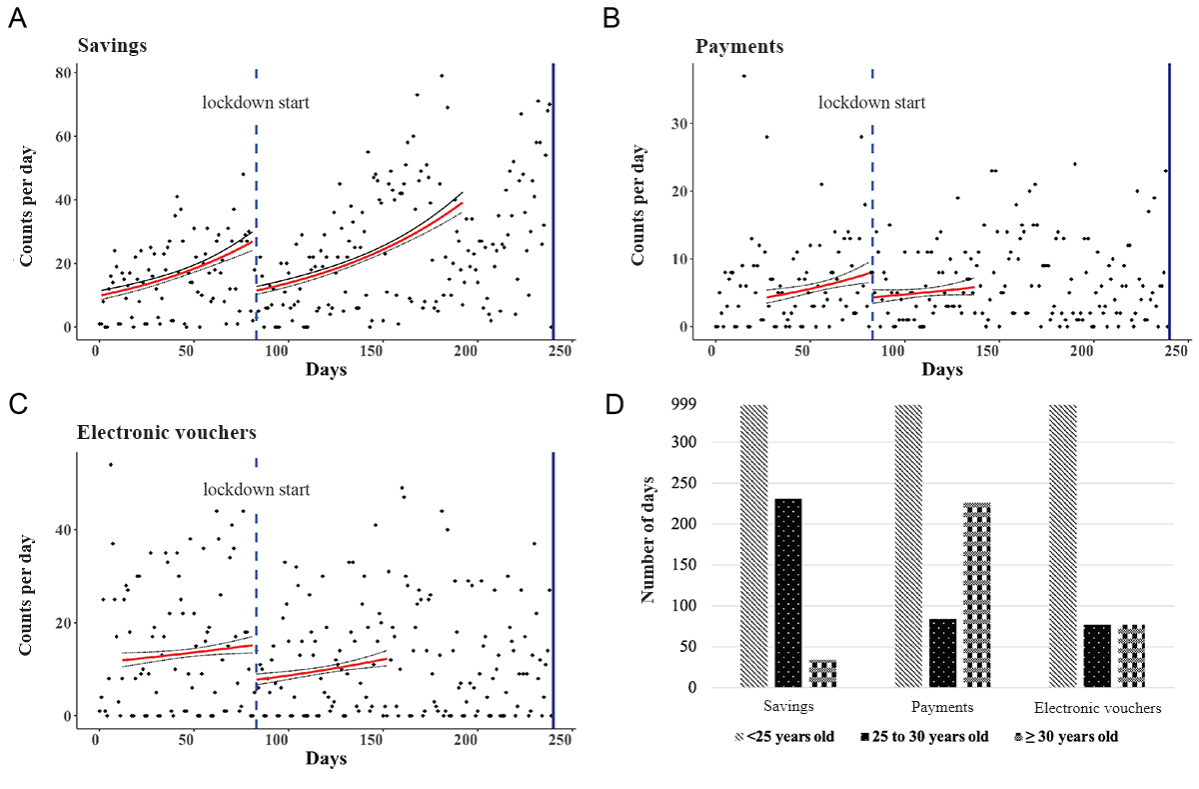
Regression discontinuity analysis of the effect of the lockdown on savings, payments, and electronic voucher use for maternal health care. (A-C) Total events of 3416 women using the mobile maternal health wallet to save and pay for maternal care at 25 public sector primary health care facilities and 4 referral hospitals in Antananarivo, Madagascar, between January 1, 2020, and August 27, 2020, depicted as counts of events per day (blue dots). The solid red line and black fine-dotted line depict the predicted incidence rate and confidence intervals, respectively, before and after the lockdown using a modified Poisson regression model and the optimal bandwidth determined by a data-dependent algorithm described by Imbens and Kalyanaraman. The origin on the x-axis corresponds to January 1, 2020. (D) The time to reach baseline for different age groups. The time relative to the lockdown for the predicted incidence rate after the lockdown to reach the extrapolated counterfactual predicted incidence rate. The time to reach the extrapolated counterfactual was the shortest in the age group of <30 years for savings and electronic voucher use and the shortest in the age group of 25-30 years for payments. In the age group of <25 years, the predicted incidence rate after the lockdown did not return to the counterfactual predicted incidence rate for savings, payments, and electronic voucher use and was coded as 999 days.

After an initial drop at the lockdown, the predicted incidence rates recovered but only crossed the extrapolated prelockdown counterfactual for electronic voucher use. Analyzing the rate of recovery separately for 3 age groups of women (<25 years, 25-30 years, and >30 years) revealed differences in the time for the predicted incidence rate after the lockdown to reach the counterfactual predicted incidence rate extrapolated from the prelockdown incidence rate (Figure 3D) [28]. For women aged <25 years, predicted incidence rates after the lockdown did not reach the counterfactual predicted incidence rates for any of the measured outcomes, indicating a lasting effect of the lockdown. For women between 25 and 30 years of age, predicted incidence rates recovered and crossed the counterfactual predicted incidence rate at 268, 84, and 77 days after the lockdown for savings, payments, and electronic voucher use, respectively. This crossing of lockdown incidence rates and counterfactual incidence rates already occurred on day 38 and day 77 for savings and electronic voucher use, respectively, in the group of women aged >30 years, indicating a faster return to prelockdown incidence rates. In the same age group, incidence rates for payments only recovered 226 days after the lockdown.

## Discussion

### Principal Findings

We aimed to quantify the effect of the COVID-19 lockdown on the use of key components of a mobile health wallet, namely, savings, payments, and electronic voucher use, for maternal health care at 25 public sector primary health care facilities and 4 reference hospitals in Antananarivo, Madagascar. Using a quasiexperimental regression discontinuity design analyzing 819,840 events for each use type, we found that mobile money–based savings, payments, and electronic voucher use for pregnancy-related health services decreased by 58.5%, 45.8%, and 49.6% at the lockdown, respectively. Our findings suggest that the lockdown had a strong negative effect on the use of the mobile health wallet. Savings quickly rebounded during the lockdown, whereas payments and voucher use were slower to recover. Further analysis of this revealed age-dependent differences in the speed of recovery. For the youngest age group of <25 years, the lockdown had a lasting effect, and none of the outcome measures returned to the prelockdown predicted incidence rates, indicating age-dependent differences in access to health care–related resources and access to health care.

To our knowledge, this is the first study quantifying the effect of the COVID-19 lockdown on the use of mobile money and electronic vouchers to access essential health services. The quasiexperimental regression discontinuity design of our study enabled causal inference on the effect of the lockdown in a real-world setting without the strong assumptions made in most observational studies. To date, there are limited published data from SSA on the effect of the lockdown on the use of maternal health care services and maternal outcomes. A cross-sectional study including all public health facilities in Rwanda found that maternal health service use indicators for antenatal care, deliveries, postnatal care, and vaccinations significantly decreased during the first national COVID-19 lockdown in March and April 2020 compared to historical controls [29]. In contrast, an interrupted time series analysis using data from 11 public sector primary health care facilities in South Africa found no change in the number of daily outpatient clinic visits for perinatal care and family planning before and after the lockdown [30]. A study using data from an ongoing nationwide birth outcomes surveillance study in Botswana found no difference in the number of facility-based deliveries during the lockdown but a slight reduction in the risk of adverse pregnancy outcomes during the lockdown compared to the prelockdown [31]. Conversely, a recent meta-analysis found a substantial effect of COVID-19 on family planning, antenatal care, institutional deliveries, and postnatal care in Ethiopia [32].

An important question is “Why did savings for maternal health care reduce during the lockdown?” The reduction in savings suggests that the lockdown diminished women’s expectations of using maternal and child health care in the future. An analysis of labor market data from South Africa during the first wave of the pandemic and a longitudinal cohort study among informal settlers in Nairobi suggested that these observations might have been caused by reduced availability of financial resources due to lockdown measures [33,34]. In South Africa, about 1 of every 3 employed people lost their jobs during the first wave of the pandemic, with women working in the informal sector being among the most severely affected [34]. Likewise, in Kenya, women were disproportionally affected by lockdown measures, resulting in increased food insecurity and lower use rates of maternal and child health care services because of financial constraints [33]. Previously, we found that the majority of pregnant women in the study area were unemployed or working in the informal sector [19], making them especially vulnerable to the economic effects of lockdowns. In addition, saving behaviors might have been affected by difficulties faced by women in converting cash into electronic currency. Survey data from Kenya, Mozambique, and Nigeria suggested that mobile money users faced difficulties depositing or withdrawing funds from cash-in/cash-out agents because agents were short on cash or closed or because of infection concerns [7].

The declines in payments and voucher use indicate a decrease in the actual use of maternal and child health care during the lockdown. An important finding from our study was that electronic voucher use, which did not require users to rely on their own financial resources to access maternal health care, reduced during the lockdown. Other studies have shown that removal of user fees is associated with increased use of maternal health care services, including antenatal care visits and facility-based normal and complicated deliveries (including cesarean sections) [35,36]. A review of demand-side financial incentive programs found increased use of antenatal care and facility-based delivery by vouchers aiming to reduce or waive the costs of care at the point of use [10]. In our study, we showed the effect of the lockdown on electronic voucher use for maternal health care services. This is an important finding because governments and implementers relied on direct cash transfers or distribution of vouchers for maternal health care services during the pandemic to ensure equitable access to care [37]. It is likely that factors contributing to the overall disruption of maternal health care services, including availability of health workers, drug supply shortage, and pandemic control measures like interrupted public transport, may also affect the uptake of voucher programs. A recent rapid review on the accessibility and use of antenatal care services in SSA found that movement restrictions, limited transport access, and anxiety about contracting COVID-19 at health facilities were the main reasons for reduced use of antenatal care services and facility-based deliveries during the COVID-19 pandemic [38]. These findings highlight the potential of digital tools, such as the MMHW, which facilitate direct communication with users, to address barriers to accessing maternal health care during lockdowns. The MMHW, for instance, allows for direct communication with users without the need for an active internet connection, using USSD and SMS text message protocols. In contrast to mass message campaigns, use of the information available through the MMHW database on users, their visits to health care facilities, and their received treatments could allow for more targeted and effective communication with users. By conveying individualized information about available transport options and emphasizing the necessity of maternal health care, these tools can help assuage concerns related to infection risks and safety measures. Ensuring that women using the MMHW are aware of public transport availability to health care facilities and providing details on schedules are crucial. Lockdowns can impose time restrictions on daily activities like grocery shopping, complicating access to antenatal and maternal care when health center opening hours coincide with restricted activity times. It is essential for women to understand that they can continue to access necessary pregnancy and maternal health care through the MMHW during lockdowns. Communication should focus on the importance of using maternal care and outlining how it can be safely accessed by adhering to social distancing guidelines and using personal protective equipment. Lastly, it is vital to ensure that women find compliance with additional procedural restrictions, such as testing and vaccination, both manageable and feasible. This can be achieved through general public engagement campaigns and the MMHW, which can send notifications, reminders, and calls to users.

Age is one of the most important determinants for accessing health care [39,40]. This has been attributed to higher financial barriers to care for younger adults, less decision-making power within a family, and lower quality of care when it can be accessed [39]. This effect is particularly pronounced for access to maternal care, including skilled assisted delivery, as young maternal age itself is associated with lower socioeconomic status and lower agency within the family, which can result in poorer health outcomes for both the mother and offspring [40]. In line with these findings, we found age-dependent differences in the effect of the lockdown on health care savings, payments, and electronic voucher use. Our findings imply that the lockdown had a stronger negative effect on expected and actual maternal care use among mothers aged <25 years, likely reflecting their comparatively lower economic standing and decision-making capacity. More research is needed to better understand the underlying mechanisms of these effects. Nonetheless, our findings highlight the importance of prioritizing support for this vulnerable demographic in efforts to mitigate the detrimental effects of lockdowns on maternal health care access.

### Limitations

There were several limitations. First, pandemic control measures in Madagascar were not consistently implemented or enforced, which limits the discriminating power of our analysis and might introduce unknown confounders that cannot be controlled. However, the stepwise implementation and partly short duration of pandemic control measures reflect the reality in many countries in SSA. To allow for a robust definition of lockdown, we chose to determine the timing and stringency based on data from the OxCGRT, which provides the most accurate data on lockdown measures for Madagascar. Second, we did not measure and include in our analysis the effects of the pandemic on the societal attitude toward health care and the perception of mobile money in general. However, these general lockdown-independent effects would likely be more gradual over a longer period and be controlled for when using a regression discontinuity analysis to infer causality. Third, participant enrollment for the main trial was ongoing during the study period; thus, the use of the mobile health wallet increased over time. To reduce confounding, we chose to report daily incidence rates per patient instead of total counts for all outcomes. Fourth, the use of the mobile health wallet may not be a substitute for the use of mobile money for health. However, because the mobile health wallet uses the mobile money infrastructure of mobile network operators, saving and payment processes for users closely resemble standard mobile money processes. Fifth, we only included data from women who used the MMHW at least once during the study period. No direct conclusions can be drawn on the change in health-seeking regarding the saving behavior of women who did not use the MMHW. However, previous studies on the user profiles of active MMHW users highlighted that users come from all sociodemographic strata [17].

### Conclusions

The lockdown significantly reduced mobile money–based savings, payments, and electronic voucher use for maternal health care. These effects on expected and actual maternal care use are crucial since many services cannot be delayed for long, with potential benefits being lost or diminished. Our results highlight the challenges in using mobile money and electronic vouchers for maternal health care services during a lockdown in a resource-restricted setting. Further research is needed to identify what specific aspects of mobile money savings, payments, and electronic voucher use caused patients to refrain from use. These findings are important for designing strategies to ensure equitable financial access to health care services during this and future pandemics.

## Appendix

Multimedia Appendix 1 Descriptive statistics.

Multimedia Appendix 2 Sensitivity analysis for different bandwidths centered at the optimal bandwidth.

## Abbreviations

IRR: incidence rate ratio
MMHW: mobile maternal health wallet
NGO: nongovernmental organization
OxCGRT: Oxford COVID-19 Government Response Tracker
SSA: sub-Saharan Africa
USSD: Unstructured Supplementary Service Data

## Data Availability

The data sets generated and analyzed during this study are not publicly available due to privacy and ethical restrictions but are available from the corresponding author upon reasonable request. The data that support the findings of this study are available with the stipulation that they will be used solely for scholarly and noncommercial purposes, in compliance with the appropriate ethical regulations. Requests for access to these data should be addressed to the corresponding author, subject to approval from the institutional review board and adherence to confidentiality agreements.
