# Supplementary material for: Effect of the COVID-19 Lockdown on Mobile Payments for Maternal Health: Regression Discontinuity Analysis
Source: JMIR Public Health Surveill. 2024 Jul 30;10:e49205. doi: 10.2196/49205 (PMC11322714; doi:10.2196/49205)
Supplement: Multimedia Appendix 1 [file publichealth_v10i1e49205_app1.docx]

**Multimedia Appendix 1.** Descriptive statistics.

| Electronic voucher - no Lockdown | | Electronic voucher - Lockdown | |
| --- | --- | --- | --- |
| Min. | 0 | Min. | 0 |
| 1st Qu. | 1 | 1st Qu. | 1 |
| Median | 12.5 | Median | 9.5 |
| Mean | 15.317 | Mean | 12.076 |
| 3rd Qu. | 25 | 3rd Qu. | 19 |
| Max. | 54 | Max. | 49 |
| IQR | 24 | IQR | 18 |

| Payments - no Lockdown | | Payments - Lockdown | |
| --- | --- | --- | --- |
| Min. | 0 | Min. | 0 |
| 1st Qu. | 1 | 1st Qu. | 2 |
| Median | 12.5 | Median | 5 |
| Mean | 15.317 | Mean | 6.476 |
| 3rd Qu. | 25 | 3rd Qu. | 9 |
| Max. | 54 | Max. | 37 |
| IQR | 24 | IQR | 7 |

| Savings - no Lockdown | | Savings - Lockdown | |
| --- | --- | --- | --- |
| Min. | 0 | Min. | 0 |
| 1st Qu. | 5 | 1st Qu. | 8 |
| Median | 15 | Median | 22 |
| Mean | 15.354 | Mean | 25.044 |
| 3rd Qu. | 23.75 | 3rd Qu. | 39 |
| Max. | 48 | Max. | 79 |
| IQR | 18.75 | IQR | 31 |
