# Supplementary material for: Effect of the COVID-19 Lockdown on Mobile Payments for Maternal Health: Regression Discontinuity Analysis
Source: JMIR Public Health Surveill. 2024 Jul 30;10:e49205. doi: 10.2196/49205 (PMC11322714; doi:10.2196/49205)
Supplement: Multimedia Appendix 2 [file publichealth_v10i1e49205_app2.docx]

**Multimedia Appendix 2.** Sensitivity analysis for different bandwidths centered at the optimal bandwidth.


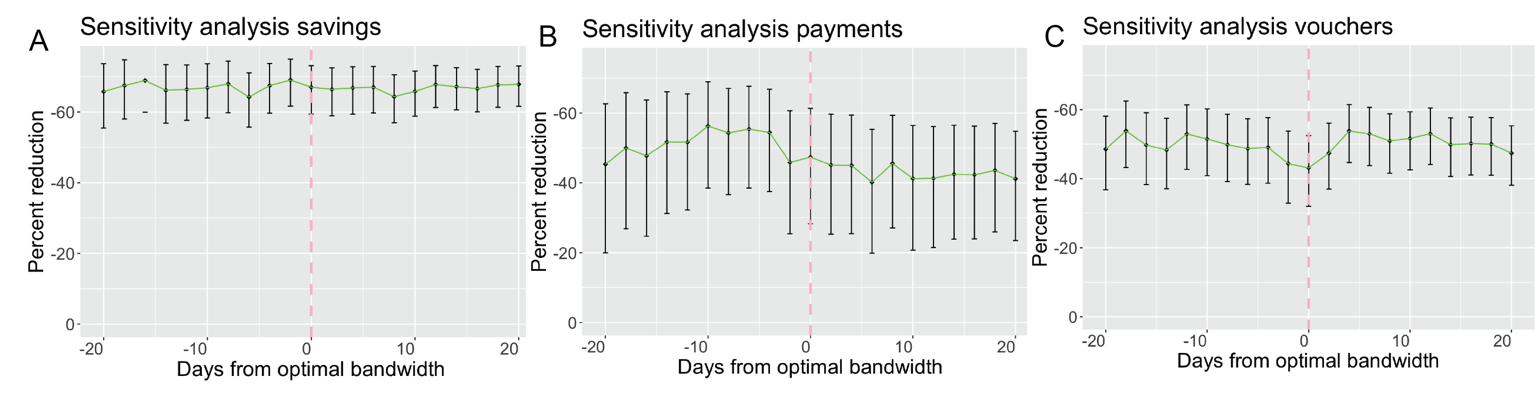


(A-C) Analysis for savings, payments and use of electronic vouchers for maternal healthcare. Pink dotted lines indicate the optimal bandwidth determined by a data-dependent algorithm. Error bars represent 95% confidence interval.
